# Experiences in reverse sequence esophagectomy: a promising alternative for esophageal cancer surgery

**DOI:** 10.1007/s00464-023-10120-y

**Published:** 2023-05-22

**Authors:** Chih-Hung Lin, Cheng-Yen Chuang, Jiunn-Liang Ko, Chung-Ping Hsu

**Affiliations:** 1 Institute of Medicine, Chung Shan Medical University, Taichung, 40201 Taiwan; 2 Division of Thoracic Surgery, Department of Surgery, Taichung Veteran General Hospital, Taichung, 40705 Taiwan; 3 Department of Medical Oncology and Chest Medicine, Chung Shan Medical University Hospital, Taichung, 40201 Taiwan; 4 School of Medicine, Tzu Chi University, Hualien, 97002 Taiwan; 5 Division of Thoracic Surgery, Department of Surgery, Buddhist Tzu Chi General Hospital, Hualien, 97002 Taiwan

**Keywords:** Esophageal cancer, Minimally invasive esophagectomy, The reserve sequence, Complication, Prognosis

## Abstract

**Objectives:**

McKeown esophagectomy is a standard and significant component of multimodality therapy in esophageal cancer, however, experience in switching the resection and reconstruction sequence in esophageal cancer surgery is not available. Here, we have retrospectively reviewed the experience of reverse sequencing procedure at our institute.

**Methods:**

We retrospectively reviewed 192 patients who had undergone minimally invasive esophagectomy (MIE) with McKeown esophagectomy between August 2008 and Dec 2015. The patient’s demographics and relevant variables were evaluated. The overall survival (OS) and disease-free survival (DFS) were analyzed.

**Results:**

Among the 192 patients, 119 (61.98%) received the reverse sequence MIE (the reverse group) and 73 patients (38.02%) received the standard operation (the standard group). Both patient groups had similar demographics. There were no inter-group differences existed in blood loss, hospital stay, conversion rate, resection margin status, operative complication, and mortality. The reverse group had shorter total operation time (469.83 ± 75.03 vs 523.63 ± 71.93, *p* < 0.001) and thoracic operation time (181.22 ± 42.79 vs 230.41 ± 51.93, *p* < 0.001). The 5-year OS and DFS for both groups were similar (44.77% and 40.53% in the reverse group vs 32.66% and 29.42% in the standard group, *p* = 0.252 and 0.261, respectively). Similar results were observed even after propensity matching.

**Conclusions:**

The reverse sequence procedure had shorter operation times, especially in the thoracic phase. The reverse sequence MIE is a safe and useful procedure when postoperative morbidity, mortality, and oncological outcomes are considered.

Esophageal cancer is the seventh most diagnosed cancer in the world in 2020. In men, it is the 6th leading cause of cancer death [1, 2]. The two most prevalent histologic subtypes are squamous cell carcinoma (SCC) and adenocarcinoma (AC). The countries of Eastern Asia have the highest regional esophageal SCC incidence rates [2]. In Taiwan, it is the 5th leading cause in male cancer death in 2019 [3].

Despite the introduction of multimodality medicines over the years to increase survival rates, such as radiotherapy, chemotherapy, and surgery, surgical resection remains the only effective treatment choice for people with resectable esophageal cancer [4–7]. There are several surgical procedures used for complete resection of the esophageal cancer. They include transhiatal esophagectomy and transthoracic techniques, such as Ivor-Lewis esophagectomy and “3-incision” McKeown esophagectomy [8–12]. The McKeown esophagectomy allows radical extended mediastinal lymph node dissection and a larger longitudinal resection margin [13].

Multiple studies have found that minimally invasive esophagectomy (MIE) is a safe and effective way to reduce perioperative morbidity and improve cancer outcomes. In the McKeown procedures, transthoracic esophagectomy is firstly performed followed by esophageal reconstruction. Although Ivor-Lewis esophagectomy is an alternative and provides more detail information regarding the intra-abdominal tumor spreading before esophagectomy, most Asian surgeons prefer McKeown esophagectomy because of its greater longitudinal safety margin.

In our institute, most esophagectomy surgeries were performed using the minimally invasive McKeown esophagectomy procedure, however, we started to reverse the resection and reconstruction procedures since 2008 [14]. Although, the initial experience was encouraging, the short- and long-term benefits of this reverse sequence procedure were sparse. Here, we reviewed our institutional experience of performing McKeown esophagectomy in MIE. We focused on the technical feasibility and safety of reverse sequence procedure in esophagectomy.

## Patients and methods

### Patient selection

We reviewed 411 patients who underwent resection for esophageal carcinoma at the Taichung Veterans General Hospital between August 2008 and December 2015 Among them, 192 patients had received MIE. All patients with a cure in mind underwent surgery. Whether to choose the standard procedure or the reverse sequence procedure is related to surgeon’s preference. The study excluded cervical cancer patients, those undergoing a conventional open-chest approach without MIE, and those undergoing surgery by a robotic operation. Those who did not have reconstructive surgery were not included in the study. Medical records were reviewed to determine the patient's age, tumor stage, type of surgery, duration of surgery, intraoperative blood loss, number of lymph nodes, radicality of resection, surgical complications, length of hospital stay, mortality, and recurrence time. The severity of postoperative complications was stratified according to the modified Clavien–Dindo classification [15].

The tumor–node–metastasis (TNM) stage was determined according to the TNM classification, 8th edition [16]. All patients were recalculated to match the TNM classification. Clinical tumor staging was based on data from endoscopic ultrasonography, abdominal sonography, computed tomography (CT), and positron emission tomography (PET). The decision to use neoadjuvant concurrent chemoradiotherapy (CCRT) was made by a multidisciplinary cancer team at the Taichung Veterans General Hospital. The treatment protocols of neoadjuvant CCRT included radiotherapy at a standard dose (50.4 Gray/28 fraction), and chemotherapy with cisplatin (at 20 mg/m^2^ for 1 h and 5-FU 800 mg/m^2^ for 24 h daily on Day 1 to 4: cycle 1, and Day 29 to 32: cycle 2). For patients receiving neoadjuvant CCRT, surgery is typically performed 4–6 weeks after the last dose of therapy [17].

The long-term follow-up of resected patients was tracked until 31 December 2021. This study was approved by the Institutional Review Board (CE19247A-2) of the Taichung Veterans General Hospital.

### Surgical procedure of the reverse sequence

#### First step (abdominal and cervical phase)

The abdominal part of the McKeown esophagectomy is typically performed first. Using laparoscopic technique, the lesser omentum space is opened by dividing the hepatogastric ligament, and the esophagogastric junction was mobilized with the esophagus looped. Dissection proceeded into the low mediastinum and then the esophagus is transected at esophagogastric junction with a stapler. The esophagus was left at the thorax and the crus muscles were closed with silk stitches. After meticulous dissection of the celiac axis and lymphadenectomy, the left gastric vessels were divided, using endoscopic linear stapler. By dividing the greater omentum with energy device and preserving the right gastroepiploic artery as feeding artery, the stomach was fully mobilized. Using linear staplers, we excised the lesser curvature side of stomach (preserve the right gastric artery), and shaped it into a narrow gastric conduit (4–6 cm in width), extracorporeally.

An oblique incision was made in the anterior border of the left sternocleidomastoid muscle. Platysma and strap muscles were divided and carotid sheath retracted. The prevertebral space entered, and the esophagus isolated. The cervical esophagus was pulled out and transected as low as possible, and the distal end of the esophagus was buried into the thorax. The retrosternal route was created by combined endoscopic and blunt dissection. Pulling up the gastric conduit to the neck through the retrosternal route, esophagogastrostomy was performed either by circular stapler or manual suturing. A 6-French silicon drain was placed around the anastomosis and the incision is closed.

#### Second step (thoracic phase)

General anesthesia with a double-lumen endobronchial tube, the thoracic phase was conducted under one lung ventilation. The camera port is inserted in the eighth intercostal space in the line of the scapular tip. The other two thoracoscopic ports were inserted at the fourth and sixth intercostal space. After dividing the upper mediastinal pleura around the esophagus, the proximal esophageal end was dragged from the cervical part directly without encircling the esophagus. Using forceps for esophageal traction, the esophagus with the meso-esophagus was dissected circumferentially. The azygos vein was encircled to allow the esophagus to pass through. The lower mediastinal pleura was divided, the distal esophageal stump was pulled out and the esophagus is dissected upward until full separation. The specimen was extracted by upsizing one port. After lymph node dissection, a 28-French chest tube was left in place posteriorly and the incisions were closed.

### Patient follow-up

Patients were examined every 3 months for the first two years, 6 months for the next two years, and annually thereafter. Tumor recurrence and death were regularly recorded following surgery. Each follow-up included a physical examination, blood analysis including SCC, chest radiography, sonogram of liver, and chest CT scan. Whenever symptoms or signs indicated a recurrence, further evaluations were performed. They used bone scintigraphy, brain magnetic resonance imaging, and/or positron emission tomography to evaluate the patient. Metastasis was classified as localized or distant. Locally recurring disease is defined as a recurrence within the esophageal bed, regional lymph nodes, or the anastomosis [18, 19]. Distant metastasis refers to metastasis that occurs at distant lymph nodes or organ sites. Overall survival was assessed from the date of surgery until the last follow-up or death. The disease-free survival was calculated from the date of surgery to the date of cancer recurrence or death from any cause.

### Statistical analyses

The data in this study were means, medians, and counts. For statistical analyses of relationships between patient group and sex, histologic type, neoadjuvant therapy, operation approach, and tumor stage, the Pearson’s Chi-square test was used. Propensity score matching was calculated based on a logistic regression model, with a caliper 0.05, matching ratio = 1:1 to balance potential bias. The two groups (standard McKeown group and reverse sequence group) were compared using the Student's t test or the Mann–Whitney *U* test, as appropriate. The Kaplan–Meier method was used to calculate the OS and DFS. The cox regression model was used to investigate the differences between groups. All comparisons were conducted two-tailed and with *p* ≤ 0.05 as the significance level. A statistical analysis was conducted using the Statistical Package for the Social Sciences (IBM SPSS version 22.0).

## Results

**Fig. 1:**
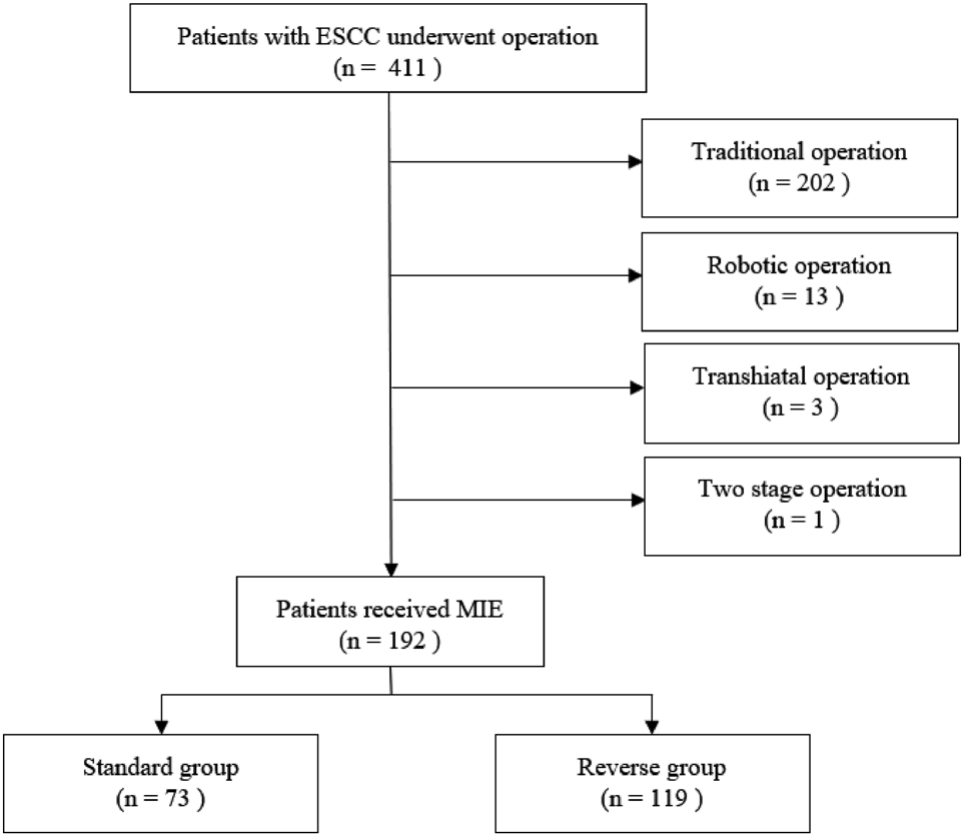
Study enrollment. Of the 192 patients who underwent surgical resection, 73 in the standard group and 119 in the reverse group

From August 2008 to December 2015, 411 patients were diagnosed as esophageal carcinoma and received surgical resection. Among them, 202 received traditional operation, 3 received transhiatal esophagectomy, 13 received robotic esophagectomy, and one had a two-stage reconstruction. The mean and median follow-up time were 3.17 years and 2.48 years (range 0.81–4.93 years) (Fig. 1).

There were no inter-group differences in terms of age, gender, tumor cell type, tumor clinical and pathologic stages (Table 1). One hundred and nineteen patients received the reverse sequence procedure and 73 patients (38.02%) received the standard procedure. Most cancers occurred at middle esophagus (70.31%). In the standard group, the proportion of upper esophageal tumors was higher than the reverse group. To eliminate the discrepancy, we use propensity score matching and the details are shown in Table 2.

**Table 1:** Patient demographic data and tumor characteristics in the two groups

	Standard (*n =* 73)	Reverse (*n =* 119)	Total (*n =* 192)	*p* value
Age, years	54.23 ± 8.23	54.91 ± 8.89	54.65 ± 8.63	0.54
Gender				0.84
Male	70 (95.89%)	112 (94.12%)	182 (94.79%)	
Female	3 (4.11%)	7 (5.88%)	10 (5.21%)	
Personal history				
Smoking	66 (90.41%)	111 (93.28%)	177 (92.19%)	0.66
Betel nut	49 (67.12%)	95 (79.83%)	144 (75.00%)	0.07
Alcohol	64 (87.67%)	113 (94.96%)	177 (92.19%)	0.12
Cell type				0.21
SCC	72 (98.63%)	111 (93.28%)	183 (95.31%)	
Adenocarcinoma	0 (0%)	6 (5.04%)	6 (3.13%)	
Adenosquamous	0 (0%)	1 (0.84%)	1 (0.52%)	
Others	1 (1.37%)	1 (0.84%)	2 (1.04%)	
Location				0.01*
Upper	10 (13.70%)	5 (4.20%)	15 (7.81%)	
Middle	49 (67.12%)	76 (63.87%)	125 (65.10%)	
Lower	14 (19.18%)	38 (31.93%)	52 (27.08%)	
Neoadjuvant	49 (67.12%)	80 (67.23%)	129 (67.19%)	> 0.99
Clinical status				
T status				0.369
cTis	3 (4.11%)	1 (0.84%)	4 (2.08%)	
cT1	4 (5.48%)	9 (7.56%)	13 (6.77%)	
cT2	8 (10.96%)	13 (10.92%)	21 (10.94%)	
cT3	57 (78.08%)	96 (80.67%)	153 (79.69%)	
cT4	1 (1.37%)	0 (0%)	1 (0.52%)	
N status				0.06
cN0	19 (26.03%)	27 (22.69%)	46 (23.96%)	
cN1	31 (42.47%)	72 (60.50%)	103 (53.65%)	
cN2	21 (28.77%)	19 (15.97%)	40 (20.83%)	
cN3	2 (2.74%)	1 (0.84%)	3 (1.56%)	
Clinical stage				0.46
0	3 (4.11%)	1 (0.84%)	4 (2.08%)	
I	4 (5.48%)	8 (6.72%)	12 (6.25%)	
II	15 (20.55%)	24 (20.17%)	39 (20.31%)	
III	48 (65.75%)	84 (70.59%)	132 (68.75%)	
IV	3 (4.11%)	2 (1.68%)	5 (2.60%)	

Continuous data were expressed mean ± SD. Categorical data were expressed number and percentage

**Table 2:** Patient demographic data and tumor characteristics after propensity score matching

	Standard (*n =* 59)	Reverse (*n =* 59)	Total (*n =* 118)	*p* value
Age, years	53.53 ± 7.56	54.41 ± 7.63	53.97 ± 7.58	0.470
Gender				1.000
Male	58 (98.31%)	58 (98.31%)	116 (98.31%)	
Female	1 (1.69%)	1 (1.69%)	2 (1.69%)	
Cell type				1.000
SCC	59 (100%)	59 (100%)	118 (100%)	
Adenocarcinoma	0 (0%)	0 (0%)	0 (0%)	
Adenosquamous	0 (0%)	0 (0%)	0 (0%)	
Others	0 (0%)	0 (0%)	0 (0%)	
Location				0.927
Upper	5 (8.48%)	4 (6.78%)	9 (7.63%)	
Middle	42 (71.19%)	42 (71.19%)	84 (71.19%)	
Lower	12 (20.34%)	13 (22.03%)	25 (21.19%)	
Neoadjuvant	40 (67.80%)	37 (62.71%)	77 (65.25%)	0.562
Clinical status				
T status				0.934
cTis	1 (1.69%)	1 (1.69%)	2 (1.69%)	
cT1	4 (6.78%)	5 (8.47%)	9 (7.63%)	
cT2	4 (6.78%)	5 (8.47%)	9 (7.63%)	
cT3	50 (84.75%)	48 (81.36%)	98 (83.05%)	
cT4	0 (0%)	0 (0%)	0 (0%)	
N status				1.000
cN0	13 (22.03%)	12 (20.34%)	25 (21.19%)	
cN1	29 (49.15%)	30 (50.85%)	59 (50.00%)	
cN2	16 (27.12%)	16 (27.12%)	32 (27.12%)	
cN3	1 (1.69%)	1 (1.69%)	2 (1.69%)	
Clinical stage				1.000
0	1 (1.69%)	1 (1.69%)	2 (1.69%)	
I	4 (6.78%)	5 (8.47%)	9 (7.63%)	
II	11 (18.64%)	10 (16.95%)	21 (17.80%)	
III	42 (71.19%)	42 (71.19%)	84 (71.19%)	
IV	1 (1.69%)	1 (1.69%)	2 (1.69%)	

Continuous data were expressed mean ± SD. Categorical data were expressed number and percentage

No significant differences existed in terms of blood loss, hospital stay, conversion rate, resection margin status, operative complication, and operative mortality (Table 3). After propensity score matching, there was a trend of having less blood loss, more operation time, less lymph node harvest, and more complication in the standard group. In subgroup analyses, we found that the operation time was shorter in the thoracic phase (Table 4). No intra-operative death had occurred in both groups. The conversion rate in the thoracic phase of the study was 2.52% (3 in 119) in the reverse group, and 2.74% (2 in 73) in the standard group.

**Table 3:** Surgical and post-operative data in two groups

	Standard (*n =* 73)	Reverse (*n =* 119)	Total (*n =* 192)	*p* value
ASA score				0.19
0	34 (46.58%)	56 (47.06%)	90 (46.88%)	
1	37 (50.68%)	63 (52.94%)	100 (52.08%)	
2	2 (2.74%)	0 (0%)	2 (1.04%)	
Hospital stay, days	17.40 ± 10.47	15.16 ± 6.85	16.01 ± 8.46	0.54
Blood loss, ml	313.08 ± 310.39	475.80 ± 676.49	413.93 ± 570.37	0.05
Operation time, minutes				
Total	523.63 ± 71.93	469.83 ± 75.03	490.29 ± 78.19	< 0.001**
Thoracic time	230.41 ± 51.93	181.22 ± 42.79	199.92 ± 52.16	< 0.001**
Abdominal time	293.22 ± 67.47	288.61 ± 66.50	290.36 ± 66.73	0.19
Lymph node number				
Total	30.08 ± 13.84	36.81 ± 15.98	34.25 ± 15.51	0.002**
Thorax	19.12 ± 11.18	21.59 ± 11.79	20.65 ± 11.59	0.08
Abdomen	10.96 ± 7.28	15.22 ± 8.32	13.60 ± 8.19	< 0.001**
Pathologic status				
T status				0.25
pT0 and pTis	29 (39.73%)	40 (33.61%)	69 (35.94%)	
pT1	8 (10.96%)	22 (18.49%)	30 (15.63%)	
pT2	7 (9.59%)	21 (17.65%)	28 (14.58%)	
pT3	28 (38.36%)	35 (29.41%)	63 (32.81%)	
pT4	1 (1.37%)	1 (0.84%)	2 (1.04%)	
N status				0.87
pN0	44 (60.27%)	69 (57.98%)	113 (58.85%)	
pN1	18 (24.66%)	31 (26.05%)	49 (25.52%)	
pN2	9 (12.33%)	13 (10.92%)	22 (11.46%)	
pN3	2 (2.74%)	6 (5.04%)	8 (4.17%)	
Pathologic stage				0.79
0	2 (2.74%)	1 (0.84%)	3 (1.56%)	
I	31 (42.47%)	49 (41.18%)	80 (41.67%)	
II	11 (15.07%)	20 (16.81%)	31 (16.15%)	
III	26 (35.62%)	41 (34.45%)	67 (34.90%)	
IV	3 (4.11%)	8 (6.72%)	11 (5.73%)	
Margin status				0.85
R0	63 (86.30%)	106 (89.08%)	169 (88.02%)	
R1	7 (9.59%)	9 (7.56%)	16 (8.33%)	
R2	3 (4.11%)	4 (3.36%)	7 (3.65%)	
Complication				0.19
Nil	52 (71.23%)	93 (78.15%)	145 (75.52%)	
Minor	12 (16.44%)	20 (16.81%)	32 (16.67%)	
Major	9 (12.33%)	6 (5.04%)	15 (7.81%)	
Hospital mortality^a^	3 (4.11%)	2 (1.68%)	5 (2.60%)	0.37
Convert	9 (12.33%)	5 (4.20%)	14 (7.29%)	0.07

*ASA* American Society of Anesthesiology. Continuous data were expressed mean ± SD. Categorical data were expressed number and percentage. ^a^Fisher’s exact test. *p < 0.05, **p < 0.01

**Table 4:** Surgical and post-operative data after propensity score matching

	Standard (*n =* 59)	Reverse (*n =* 59)	Total (*n =* 118)	*p* value
ASA score				0.965
0	29 (49.15%)	28 (47.46%)	57 (48.31%)	
1	30 (50.85%)	31 (52.54%)	61 (51.69%)	
Hospital stay, days	17.17 ± 10.88	15.37 ± 6.36	16.27 ± 8.92	0.750
Blood loss, ml	297.63 ± 332.81	516.61 ± 433.15	455.08 ± 299.18	0.003**
Operation time, minutes				
Total	525.85 ± 73.14	474.58 ± 78.63	500.21 ± 79.87	< 0.001**
Thoracic time	228.81 ± 48.89	177.97 ± 46.86	203.39 ± 54.08	< 0.001**
Abdominal time	297.03 ± 69.69	296.61 ± 69.71	296.82 ± 69.40	0.466
Lymph node number				
Total	30.58 ± 14.19	36.27 ± 15.14	33.42 ± 14.89	0.035*
Thorax	19.88 ± 11.09	21.44 ± 11.44	20.64 ± 11.24	0.395
Abdomen	10.69 ± 7.42	14.86 ± 8.35	12.78 ± 8.14	0.003**
Pathologic status				
T status				0.418
pT0 and pTis	25 (42.37%)	17 (28.81%)	42 (35.59%)	
pT1	6 (10.17%)	11 (18.64%)	17 (14.41%)	
pT2	7 (11.86%)	11 (18.64%)	18 (15.25%)	
pT3	20 (33.90%)	19 (32.20%)	39 (33.05%)	
pT4	1 (1.69%)	1 (1.69%)	2 (1.69%)	
N status				0.969
pN0	36 (61.02%)	37 (62.71%)	73 (61.86%)	
pN1	16 (27.12%)	14 (23.73%)	30 (25.42%)	
pN2	6 (10.17%)	6 (10.17%)	12 (10.17%)	
pN3	1 (1.69%)	2 (3.39%)	3 (2.54%)	
Pathologic stage				0.498
0	1 (1.69%)	1 (1.69%)	2 (1.69%)	
I	28 (47.46%)	23 (38.98%)	51 (43.22%)	
II	7 (11.86%)	13 (22.03%)	20 (16.95%)	
III	21 (35.59%)	18 (30.51%)	39 (33.05%)	
IV	2 (3.39%)	4 (6.78%)	6 (5.08%)	
Margin status				0.625
R0	52 (88.14%)	49 (83.05%)	101 (85.59%)	
R1	4 (6.78%)	7 (11.86%)	11 (9.32%)	
R2	3 (5.08%)	3 (5.08%)	6 (5.08%)	
Complication				0.042*
Nil	43 (72.88%)	46 (77.97%)	89 (75.42%)	
Minor	8 (13.56%)	12 (20.34%)	20 (16.95%)	
Major	8 (13.56%)	1 (1.69%)	9 (7.63%)	
Hospital mortality^a^	3 (5.08%)	0 (0%)	3 (2.54%)	0.244
Convert	6 (10.17%)	2 (3.39%)	8 (6.78%)	0.272

*ASA* American Society of Anesthesiology. Continuous data were expressed mean ± SD. Categorical data were expressed number and percentage. ^a^Fisher’s exact test. *p < 0.05, **p < 0.01

### Lymph node status

In terms of the total number of lymph nodes, the reverse group was higher than the standard group (36.81 ± 15.98 vs 30.08 ± 13.84, *p* = 0.002). The lymph node number was higher mainly in the abdominal part of the body. After propensity score matching, the reverse group still harvested more lymph nodes.

### Prognosis

**Fig. 2:**
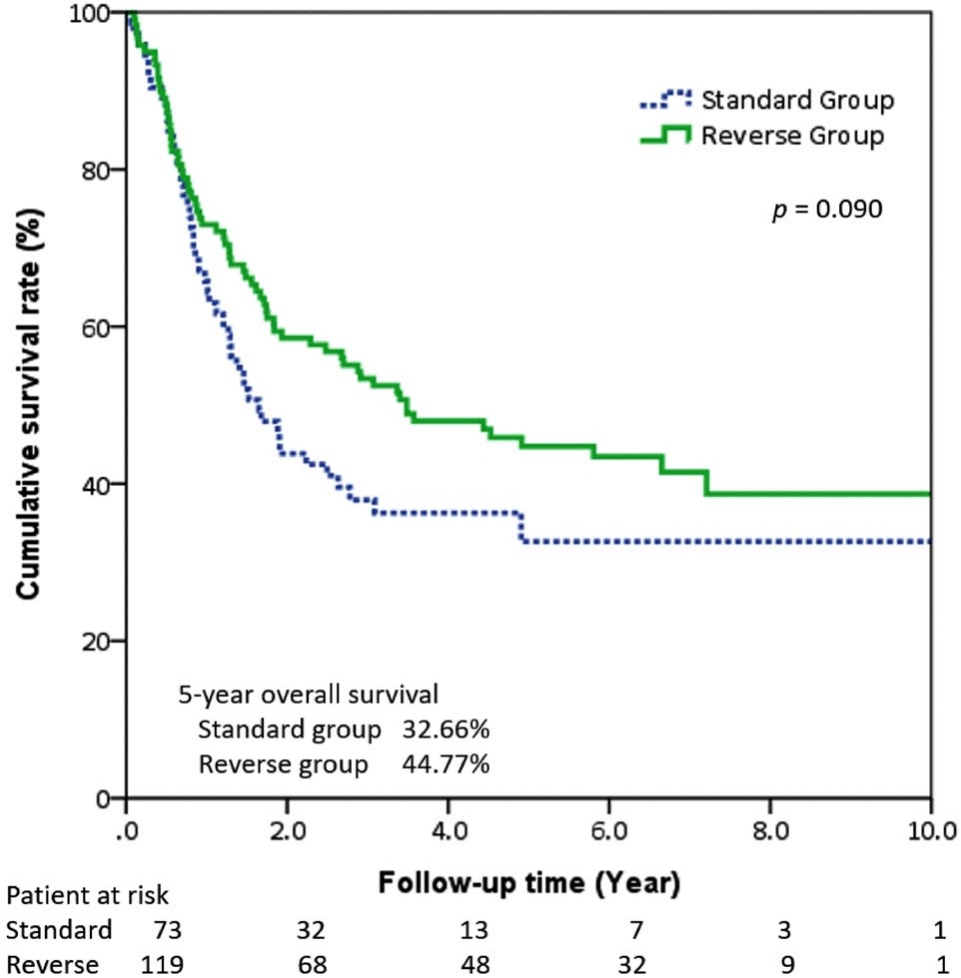
The Kaplan–Meier curves showed the overall survival for all stage in two groups

**Fig. 3:**
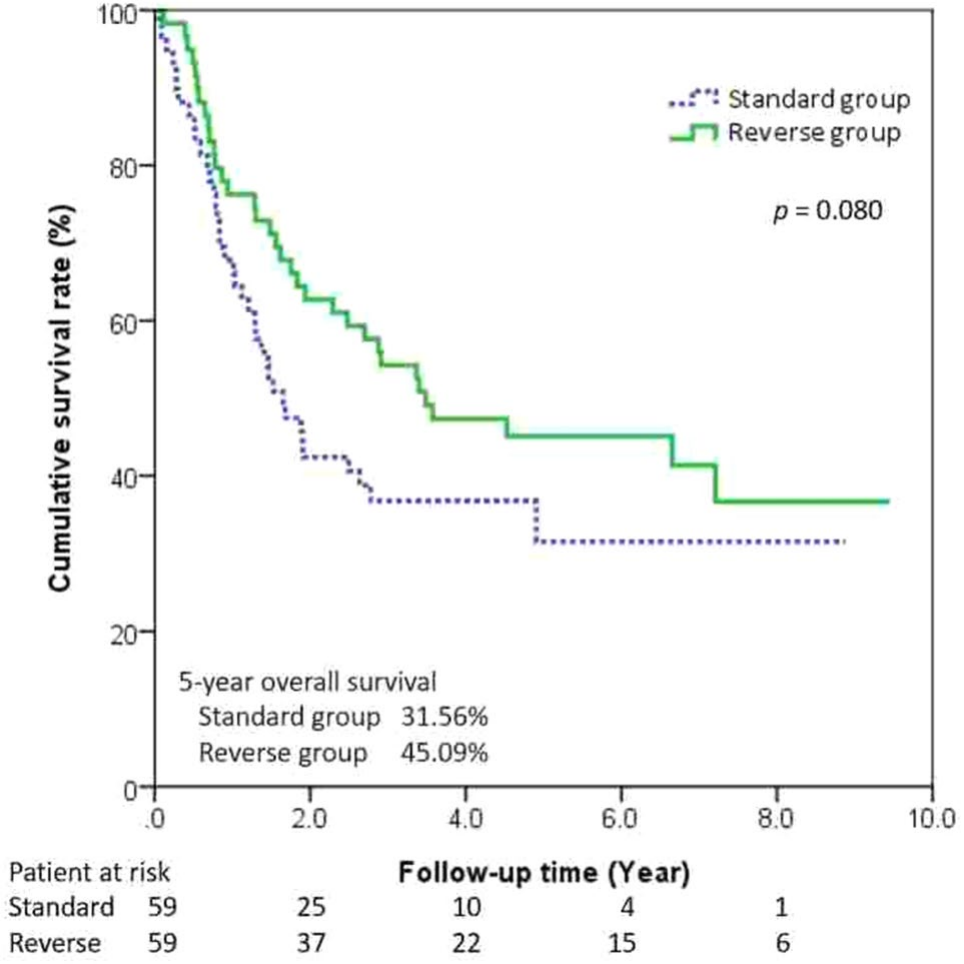
The Kaplan–Meier curves showed the overall survival for all stage after propensity score matching

**Fig. 4:**
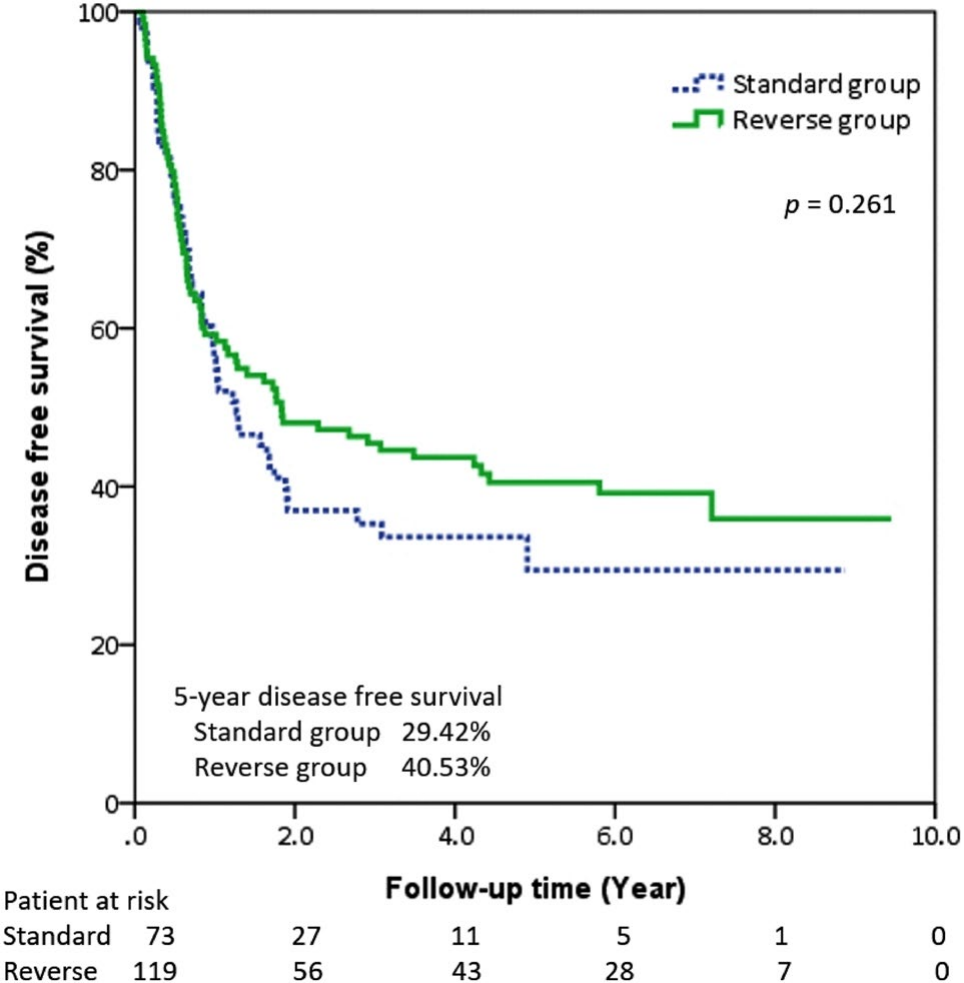
The Kaplan–Meier curves showed the disease-free survival for all stage in two groups

**Fig. 5:**
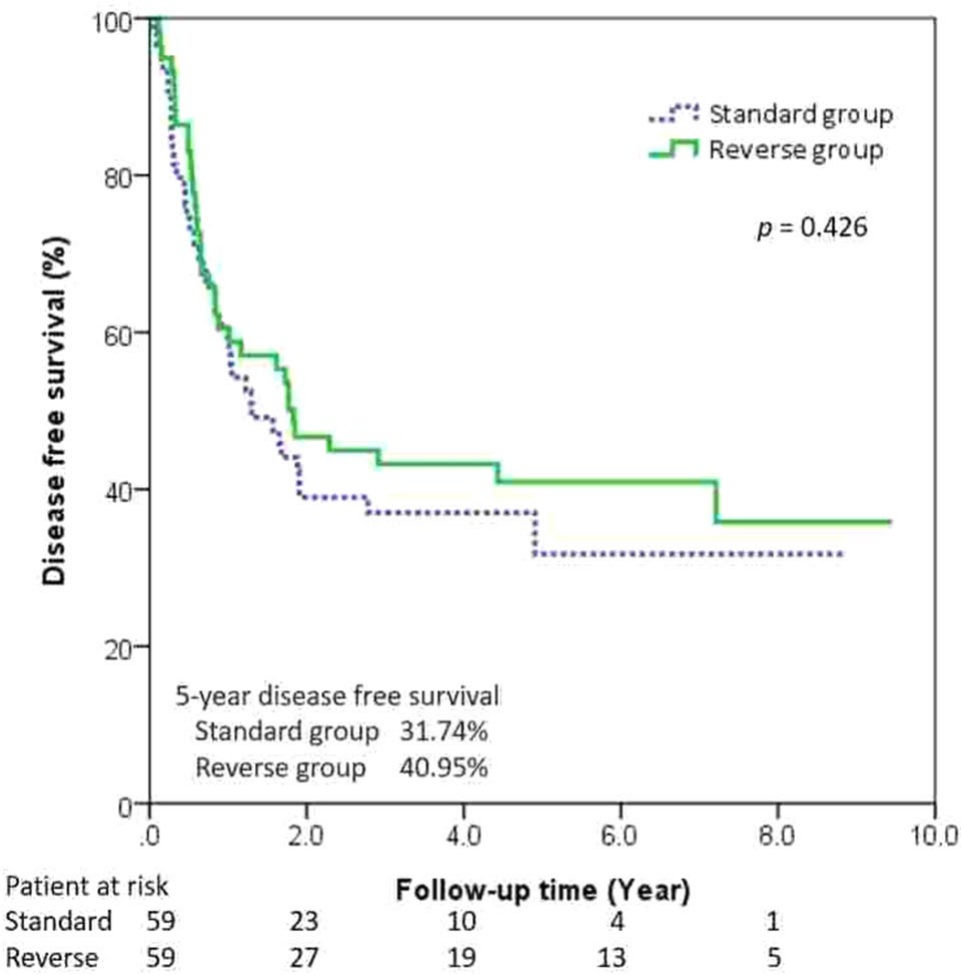
The Kaplan–Meier curves showed the disease-free survival for all stage after propensity score matching

Tumor-free margins were seen in 89.08% of the patients in the reverse group (*n =* 106) as well as in 86.30% of those in the standard group (*p* = 0.73). During follow-ups, 65 patients had tumor recurrence (Table 5). They were all involving the circumferential margin except 1 patient in the reverse group (i.e., carcinoma in situ in the proximal margin). There was no inter-group difference in the time to recurrence between the two groups. Multivariate analysis of the 2 significant variables determined by univariate analysis identified that pathologic N status independently impacted on survival in our study, not the procedure sequence (Table 6). The 5-year OS was 44.77% in the reversed group and 32.66% in standard group, with no statistical significance when examining overall survival according to overall stages and propensity score matching (Figs. 2 and 3). There was no significant difference between the 5-year DFS rates for the reverse group and the standard group (Figs. 4 and 5).

**Table 5:** Recurrence data before and after propensity score matching

Outcome	Before propensity score matching			After propensity score matching		
	Standard (*n =* 73)	Reverse (*n =* 119)	*p* value	Standard (*n =* 59)	Reverse (*n =* 59)	*p* value
Recurrence	19 (26.03%)	46(38.66%)	0.09	15 (25.42%)	26 (44.07%)	0.033*
Recurrence type			0.28			0.055
Locoregional	6 (8.22%)	22 (18.49%)		4(6.78%)	15 (25.42%)	
Distal	13 (17.81%)	24 (20.17%)		11(18.64%)	11 (18.64%)	
Recurrence-free interval, month (median)	15.24(6.48–39.6)	21.24(6.24–69.6)	0.12	15.58 (5.64–42.72)	21.24 (6.96–67.32)	0.166

* *p* < 0.05, ** *p* < 0.01

**Table 6:** Univariate and multivariate Cox proportional hazard model for survival

	Univariate analysis				Multivariate analysis			
	HR	95%CI		*p* value	HR	95%CI	*p* value	
Age (years)	1.03	(1.00–1.06)		0.057				
Gender								
Male	Reference							
Female	1.89	(0.46–7.76)		0.377				
Procedure								
Standard	Reference							
Reverse sequence	0.67	(0.42–1.08)		0.098				
Location								
Upper	Reference							
Middle	0.81	(0.35–1.90)		0.633				
Lower	0.77	(0.30–1.98)		0.584				
Neoadjuvant therapy	1.61	(0.96–2.70)		0.071				
Pathologic status								
pT1	Reference							
pT2	0.96	(0.48–1.92)		0.925				
pT3	1.44	(0.86–2.41)		0.158				
pT4	3.43	(0.80–14.63)		0.096				
pN0	Reference				Reference			
pN1	2.12	(1.26–3.57)		0.004**	2.065	(1.21–3.51)	0.007**	
pN2	2.54	(1.21–5.32)		0.013*	2.407	(1.14–5.07)	0.021**	
pN3	4.29	(1.30–14.18)		0.017*	4.568	(1.37–15.17)	0.013**	
Resection margin status								
R0	Reference				Reference			
R1	1.47	(0.70–3.09)		0.306	1.230	(0.57–2.62)	0.593	
R2	2.56	(1.02–6.42)		0.046*	2.439	(0.96–6.18)	0.060	

*HR* hazard ratio. * *p* < 0.05, ** *p* < 0.01

## Discussion

Results confirmed the feasibility, safety, and advantage of the reverse sequence procedure in esophagectomy for selected patients undergoing MIE.

In 1913, Franz Torek performed the world’s first successful subtotal thoracic esophagectomy for a patient with esophageal carcinoma [20]. To improve proximal margins, McKeown introduced the 3-incision procedure which has become one of the mainstreams of standard procedures [10, 12, 13]. With instrumental and technical improvement over time, minimally invasive surgery is the current trend. However, a learning curve is needed to familiarize with the minimally invasive surgery [21–23]. In the early period of this study, we try every effort to perfecting the operation procedure, and the results are likely reflected in longer average surgical time.

In the transhiatal esophagectomy and Ivor-Lewis esophagectomy, the abdominal part is approached first. Clinically, esophageal SCC mostly occurs in the thoracic cavity. Even without neoadjuvant CCRT, the bulky tumor in the thoracic cavity may block the surgical field. Although many studies showed that the post-neoadjuvant inflammatory responses and dense fibrosis at the site of the primary tumor or surrounding lymph nodes do not increase complication rates, [24, 25] they do increase the complexity of esophageal surgery, especially for junior surgeons. In our study, tumors at upper-third were 3 times more in the standard group. This location is well known to be the most difficult location and thus more time-consuming to dissect and therefor may in part account for the longer thoracic time in the standard group. However, we still could find similar result after propensity score matching. Therefore, the timesaving was mainly due to the reverse sequence procedure.

In the reverse sequence procedure, we could approach the tumor from the relative-normal tissues in the cervical and abdominal regions. Pre-divided upper and lower esophageal ends provides more degree of freedom in performing esophageal dissection which makes esophagectomy become easier. In the thoracic phase, we could drag the esophagus from both stumps directly without encircling the esophagus and this avoids initial countering of the difficulties when dissecting/looping the cancer bearing segment or irradiation induced fibrotic change in the thorax. This may not make a difference to experienced surgeons but make sense to junior surgeons. Therefore, the shorter operation time may present that the reverse procedure was an easier familiar approach for surgeons. Besides, the reverse sequence did not increase the abdominal operation time while significantly shortening thoracic operation time.

Wang and his colleague [26] reviewed 735 patients who received MIE for esophageal SCC, and reported a 33% of overall 5-year survival in stage III. Similar findings to ours were found in other reports [27–30]. In our series, positive resection margins were found in 10.92% of patients in the reverse group, and 13.70% in the standard group. There was no local recurrence at anastomosis in both groups. These radicality rates were consistent with other studies [27, 31, 32]. According to our study, the reverse sequence did not compromise oncological outcomes, though slightly increased locoregional recurrence was observed, their 5-year OS and DFS were superior to those of the standard group after propensity score matching, which may be due to higher distal recurrence of the standard group. Furthermore, a greater harvest of lymph nodes in the reverse group, especially in the abdominal part, was noted. The reverse MIE procedure were mainly performed by two of our group (Hsu CP and Lin CH) and this may count for the discrepancy. The difference in numbers of intra-abdominal LN retrieved persists even after propensity score matching (*p* = 0.003), however, the distribution of pN status were similar after propensity score matching (*p* = 0.969).

No significant differences in postoperative complications were observed between the two groups. However, our complications were relatively slightly higher compared with other studies [33–35]. The most common complication was anastomotic leakage. The leakage rate was 16.44% in the standard group and 16.81% in the reverse group. The leakage rate was comparable to other research [36]. According to the previous studies, [33, 35, 37] cervical anastomosis has a higher leakage rate, and all our patients had cervical anastomosis. Thus, we routinely performed a feeding jejunostomy. In addition, once leakage was suspected, even presented only as a mild turbid discharge, the cervical wound was opened immediately for drainage. Therefore, our anastomotic leakages were all type I without the need for intervention or surgery [38]. The infection was rapidly controlled with adequate drainage without prolonging hospitalization.

There are several pros of the reverse sequence procedure. Firstly, the intra-abdominal condition can be meticulously evaluated laparoscopically to prevent unnecessary esophagectomy or we can shift to Ivor-Lewis esophagectomy as a salvage procedure, immediately; Secondly, pre-divided upper and lower esophageal ends provides more degree of freedom in performing esophageal dissection which makes esophagectomy become easier; Thirdly, it provides a more friendly circumstance for the trainee to perform esophagectomy.

On the other hand, potential cons include intra-thoracic tumor spreading (though not observed in this series), and the posterior mediastinal route is not possible for gastric conduit pull-up.

There are some limitations to our study. First, this is a retrospective study at a single institute. Propensity score matching was used to identify a balanced cohort, however, selection bias was unavoidable as the reverse MIE procedure were mainly performed by two surgeons. The number of patients was still not enough. Second, although we had five-year OS and DFS, the follow-up period was still limited. Third, our institute started MIE in 2008. In the early stages, we were on the learning curve of MIE and therefore the average operation time and blood loss were higher. However, this study included and analyzed all data within two groups for the entire period. Fourth, neoadjuvant therapy was not commonly used in our institute until 2010. Although there were no significant differences between the two groups, occult effects should still be noted. At last, most of our patients had squamous cell carcinoma. This procedure is still being investigated as a possible treatment option for all patients with esophageal cancer.

## Conclusions

The reverse sequence MIE procedure had shorter operation times, especially in the thoracic phase. It is a safe and useful procedure when postoperative morbidity, mortality, and oncological outcomes are considered. We recommend this procedure as a promising alternative for esophageal cancer surgery.

## Abbreviations

OS: Overall survival
DFS: Disease-free survival
SCC: Squamous cell carcinoma
AC: Adenocarcinoma
MIE: Minimally invasive esophagectomy
TNM: Tumor–node–metastasis
CT: Computed tomography
PET: Positron emission tomography
CCRT: Concurrent chemoradiotherapy
